# Implementation, experience, and challenges of urban health extension program in Addis Ababa: a case study from Ethiopia

**DOI:** 10.1186/s12889-021-10221-0

**Published:** 2021-01-19

**Authors:** Getahun Zebre, Abraham Tamirat Gizaw, Kasahun Girma Tareke, Yohannes Kebede Lemu

**Affiliations:** 1 Department of Epidemiology, Institute of Health, ABH-Jimma University, P.O.BOX 378, Jimma, Ethiopia; 2 Department of Health, Behavior, and Society, Institute of Health, Jimma University, P.O.BOX 378, Jimma, Ethiopia

**Keywords:** Urban health extension program, Implementation, Challenges, Qualitative case study, Ethiopia

## Abstract

**Background:**

Even though the urban health extension program (HEP) has been implemented since 2009, little was known about its implementation, experience and challenges. Therefore, this study was aimed at exploring the implementation, experience, and challenges of the urban HEP.

**Methods:**

A qualitative case study was conducted in Addis Ababa from November 15 to December 29, 2017. The study participants were recruited purposefully. The parent populations were health extension professionals (HEPs). However, health post supervisors, health development army leaders (HDAs), Addis Ababa city HEP administrators, and other community members were also involved in the study. Four focus group discussions and 31 in-depth and key informant interviews were conducted. Data were transcribed verbatim, translated into the English, and analyzed by an inductive thematic analysis approach using Atlas ti7.1 software.

**Result:**

The study found that there were 15 health service packages of the urban HEP delivered to the community based on the need of the households. The strategies for the program implementation were provision of trainings, home visitation, creation of model households, strengthening of HDAs, supervision and reporting, referral and feedback, and social and community mobilization. However, program implementation was challenged by the health system related challenges (health service package and delivery, workload of HEPs, shortage of trained HEPs, lack of regular supervision or monitoring, lack of logistical or motivational support, poor supply chain management, dissatisfaction of HEPs, assigning of more than expected households for HEPs, etc.), multisectoral related and community related challenges (HDAs need of incentives, and lack of graduating model households as per the plan, etc.).

**Conclusions:**

Although the program had a significant contribution to the health of community, it was affected by different challenges that underscore the need to develop different strategies and taking of actions. Therefore, the district health office, health centers and stakeholders from different sectors should have to support and motivate the HEPs and HDAs, and work together with them for successful implementation of the program.

**Supplementary Information:**

The online version contains supplementary material available at 10.1186/s12889-021-10221-0.

## Background

Ethiopia has been implemented a successive Health Sector Development Plan since 1997 and has made huge strides in improving access to health services and improvements in health outcomes. The country’s health indicators have been remarkably improved from one of the worst in Sub-Saharan Africa to amongst the standout performers in two decades [1]. Priority given to the Health Extension Program (HEP) was the central feature for this progressive and remarkable achievement, through which cost-effective basic health services are being delivered equitably for all community [2]. The program was adopted to achieve a universal primary health care coverage [3].

The program was initially launched in 2003 to enhance preventive and promotive health care aspects in four agrarian regions (i.e., rural population) through delivering 16 health extension packages under four thematic areas: Hygiene and environmental sanitation; disease prevention and control; family health services; and health education and communication. However, due to the importance of preventive and promotive health services for pastoral and urban communities too, and since most of the country’s health problems are attributed to preventable infectious and communicable diseases, the program was expanded to these segment populations in 2006 and 2009, respectively [3]. The rural HEP is implemented by health extension workers (HEWs), while the urban health extension program (UHEP) is implemented by urban HEWs, named as Health Extension Professionals (HEP). These professionals are clinical nurses (i.e., diploma) and who taken a three months pre-service training on the basics of HEP and packages [4, 5].

The UHEP has an essential health service packages grouped into four core preventive health services: Hygiene and environmental sanitation, disease prevention and control, family health services and accident prevention and first aid. The program targets to the wellbeing of urban populations and it is deeply rooted in communities, providing primary level prevention activities to household members and encouraging them to be responsible for their health [3, 6]. To deliver the services to community members, one urban HEP is assigned to serve 500 households, and they are expected to spend 75% of their time undertaking home to home or outreach activities [4]. The health developmental armies (HDAs) also support the HEPs to deliver the service packages, and are playing a significant role in creating the required awareness and demand of health and healthcare, empowering the community to ensure the continuity and sustainability of health programs, regulation of their respective local health facilities and community health interventions [1, 7].

Despite this fact, little is known about the program implementation and community’s perspectives on the program. Thus, it was worth to conduct a study to fill these research gaps, and obtain input for better implementation. Therefore, this study was employed a qualitative case study to explore the implementation, strategies, experiences and challenges for the successful implementation of UHEP in Addis Ababa.

## Methods

### The Ethiopian health care system

Currently, Ethiopia follows a three-tier health care system: primary, secondary and tertiary levels of care. At the grass root level, there is a primary level of care (PHC) which includes primary hospitals, health centers and health posts. A primary hospital provides emergency, inpatient and ambulatory services, and referral site for health centers; totally provide health care service for an average population of 100, 000. Also, it acts as a practical training center for nurses and other paramedical health professionals. The secondary health care system includes a general hospital that act as a referral center for primary hospitals, and also training center for health officers, nurses and emergency surgeons. A tertiary health care system includes a specialized hospital, a referral center for general hospitals. Under primary health care level, there is a primary health care unit (PHCU) which comprises one referral health center and five satellite health posts. The health posts are the lowest-level health system facility, and the point where PHC is administered and primary services facilitated. Therefore, the HEP is a program designed to provide the primary health care services at the nearby community [7–9].

### Study design, setting, and period

A qualitative case study was conducted in Addis Ababa, the capital city of Ethiopia, from 15 November – 29 December 2017. The city was divided into 10 sub-cities and 116 districts, and according to the 2007 population census, there were a total of 3,384,569 populations with an annual growth rate of 3.8%. There were 14 Government Hospitals, 13 private hospitals and 96 functional health centers that provides the health care service for the community members. Additionally, there were 1288 urban HEPs who deliver the HEP for the community members [6].

### Study participant and participant recruitment

A purposive sampling technique was used to recruit study participants from five sub-cities. The sub-cities were selected purposefully considering the population number and the number of districts. The districts were selected based on the maximum variation sampling technique with their previous year program performance evaluation; one of the top performers and the least performer were selected from each selected sub-cities. The parent populations in this study were the urban HEPs. However, to increase and enhance the richness of data, the urban HEP supervisors, Health Development Army leaders, Addis Ababa city HEP administrators, and other community members were recruited and included in the study.

### Data collection instrument and procedures

Data were collected using a semi-structured questionnaire (Supplementary file 1)**,** which was first developed in English and then translated into Amharic language and back-translated for consistency. It was developed in relation to the research questions while taking into account local knowledge and cultural sensitivities (i.e., How does the urban HEP implementation is being implemented? What strategies are being used to implement the urban HEP? What experiences do you have on the implementation of urban HEP? What challenges have you faced during the implementation of urban HEP?). The sequence of the questions moved from the more general to the specific.

The data were collected through in-depth interview (IDI), key informant interview (KII) and focused group discussion (FGD). A total of sixty-two diversified participants were involved in the study. Ten IDIs were conducted with the parent populations (urban HEPs). The KIIs were conducted with key informants, and ten KIIs were conducted with women from modeled and non-modeled households (5 from each group), five KIIs with HDAs, five KIIs with Urban HEP supervisors, and one KII with Addis Ababa city urban HEP administrator.

In addition, four FGDs were conducted involving different groups of community members. In each FGD group, 7–9 individuals were participants. The participants were women from the model family training, women from model family household and non-model family household, husbands from model family household and non-model family household, pregnant women, women who gave birth within six months prior to data collection, and other reproductive age group peoples.

Two interviewers [i.e., principal investigator-GZ and research assistant) who have an educational background in health science and previous experience of conducting the qualitative study participated in the data collection. The IDIs and KIIs were conducted between the principal investigator (modulator) and study participant. However, the FGDs were conducted together with the research assistant who taken notes and recorded voices of the participants. Data were collected at the participants’ natural setting, given that interviews conducted with women and HDAs in their respective home; interview conducted by the urban HEPs, their supervisors and city urban HEP administrator at their respective office, and the focus group discussions within the community. The interviews lasted from 50:15–60:00 min, and the FGDs lasted from 1:43–2:15 h.

### Trustworthiness

To ensure trustworthiness of the study findings, different techniques were used. First, a semi-structure guide was developed in relation to the research questions, and pretested at other districts within the study setting. Second, the principal investigator was practiced on how to pose and probe questions, listen, and record participants’ responses at that time. Third, the principal investigator spent enough time at the study setting, communicating with UHEPs and key informants like the HEPs supervisors, and observed some contexts that was reported in this study. Forth, adequate and appropriate time was taken during the interview [approximately for an hour] and the FGDs [more than one and a half hours] to discuss major thematic areas. Fifth, data debriefing was conducted with the research assistant on a daily basis to discuss key findings, identify data saturation and refine lines of inquiry. Sixth, method triangulation (i.e., collecting data through three approaches- IDI, KII and FGD), and participant triangulation (collecting data from diversified group of participants- including the perspective of different stakeholders) were used to triangulate the data. Seventh, the context of the study setting, a diversity of participants, data collection processes and analysis were thickly described. Eighth, data were audiotaped and precisely and consistently coded using codebook manual. Ninth, the transcripts and the findings with were shared to the UHEPs and some of key informants for member checking. Then, explicit consideration and discussion of discrepant interpretations were discussed and resolved through negotiation. The tenth, the interpretations and findings of the study were audited, verified and confirmed by an external auditor who has experience in qualitative research.

### Data analysis

The data were analyzed through an inductive thematic analysis approach; given that the codes, categories and themes were generated from the data. Analysis was carried out simultaneously with data collection. Data were debriefed on a daily basis with the research assistant to ensure data saturation, and completeness and consistency with the simultaneous incorporation of data from field note. The principal investigator and research assistant conducted verbatim transcription of the data (i.e., data were collected in Amharic) from audio-recorded material. Then, ensuring the completeness and consistency of transcriptions, the data were translated to English. Translated data were cross-checked for completeness and consistency in meaning with the transcriptions.

Then, reading and re-reading of the transcript were done to extract important concepts related to the research objectives, and get a sense of wholeness. After that, transcripts with rich data were selected and line by line coding was conducted separately on the ATLAS.Ti.7.1 software package. Checking for inter-coder consistency, code book manual was developed to precisely define codes to ensure code consistency and credibility. Then, using the developed codebook manual, the whole data were coded by the principal investigator. Potential categories and themes were developed by clustering sub-categories and categories, respectively, which answers the research question. The coding system was repeated two times while refining the codebook, categories and themes. Finally, the findings were presented with major theme, categories, sub-categories and quotations derived from the data.

## Results

### Socio-demographic characteristics

The total 62 individuals were participated in the study. The majority of them were married females, and age ranged from 31 to 40 years old. Also, the majority of health professionals had work experience of four years and above [Table 1].

**Table 1:** Socio-demographic characteristic of participants who take part in the study, Addis Ababa, 2017

Participant characteristic		N (%)	Participants characteristic		N (%)
Sex	Male	17 (27.4)	Educational status	Primary	10 (16.1)
	Female	45 (72.6)		Secondary School	16 (25.8)
Age	18–30 years	11 (17.7)		Preparatory school	13 (21)
	31–40 years	42 (67.4)		Diploma	10 (16.1)
	≥ 41 years	9 (14.5)		University	13 (21)
Marital status	Single	20 (32.3)	Work Experience*n = 21	1/2–2 years	4 (19)
	Married	38 (61.3)		2–3 years	3 (14.3)
	Divorced	3 (4.8)		3–4 years	3 (14.3)
	Widowed	1 (1.6)		≥ 4 years	11 (52.4)

*Urban HEPs and their supervisors, Addis Ababa city HEP administrator, and HDAs

The findings of this study were organized into three major themes, ten categories and fifteen sub-categories. The major themes were implementation of urban HEP and program fidelity, strategies used to facilitate the implementation of the Urban HEP (i.e., training, home visit, creation of model households, strengthening of HDAs, supervision and monitoring, referral and feedback, and social and community mobilization), and the challenges for the successful implementation of urban HEP (health system, multisectoral and community related challenges). All these themes were described as follows.

### The implementation of urban HEP and program fidelity

Across the interviews and group discussions, participants mentioned that the urban HEP was launched and practically the implementation started in Addis Ababa on July 2008 and September 2009, respectively. They indicated that there were 15 essential health care packages delivered to the community under four major program areas (Table 2). It was also mentioned that the packages were delivered based on the household needs. For example, one urban HEP mentioned that they were providing 15 packages, but if the need of the household was to take training on family health service, intense training would be given to them. However, the HEPs and supervisors mentioned that even though all the packages were being implemented, there was a need to work hard on sanitation, hygiene and non-communicable disease, given that in the context of the city, there was poor waste handling and management at households and society level, and also increased burden of non-communicable diseases.

*“We provide all the fifteen packages of the health extension program focusing on the need of households. For example, if households need to take basic training on family health services, we provide intense training about it.” (35–40 year’s old, female, HEPs, IDI)*

*“Most of the components of the packages are being delivered to the community. However, much effort is needed on sanitation, hygiene and non-communicable disease. This is because, in the city, there is poor waste handling or management both at a household level and as a society.” (30–35 years old, male, HEPs supervisor, IDI)*

It was also mentioned that community members had got important information on disease prevention and control, and there were an improvement in their knowledge and behavior towards the prevention of both communicable and non-communicable diseases.

*“The health extension professionals teach us how to keep our and children’s hygiene and health”. (30–35 years old, female, community members, IDI)*

Regarding family health, in all of the Districts, it was mentioned that regular immunization, family planning and promotion of maternal and child health care services were provided to the community by the HEPs.

*“All pregnant women in our community have a follow-up at health centers. After delivery, our children get vaccine on time. The health extension professional teaches us regularly about family planning and the importance of vaccination.” (45–50 years old, female, community member, IDI)*

The participants also mentioned that the urban HEP supported the households to have a hand washing facility in nearby the latrines, prepare separate liquid waste disposal pits, had clean cooking practices, kept drinking water safe and free from contamination, and kept hygiene and sanitation of the environment. On the other hand, they mentioned that, in some districts, there were no first aid or emergency package trainings provided to the community due to lack of equipment, knowledge or skill.

*“We didn’t provide first aid or emergency trainings regularly due to lack of materials or equipment.” (30–35 years old, female, HEPs, IDI)*

**Table 2:** The Health Service Packages provided to the community members through urban HEP in Addis Ababa, 2017

Major program areas	Essential services provided under each major area
Hygiene and environmental sanitation	(1) Solid and liquid waste disposal (2) Personal hygiene and healthy home environment (3) Food and water safety (4) Latrine construction and utilization
Disease prevention and control	(1) Malaria (2) TB and leprosy (3) HIV and AIDS (4) Non communicable disease (5) Mental illness
Family health services	(1) Maternal and child health, (2) Family planning, (3) Immunization, (4) Youth and adolescent health, and (5) Nutrition.
Accident prevention, control and first aid, and referral services	

### Strategies used to facilitate the implementation of urban HEP

#### Training

Study participants mentioned that the urban HEPs were diploma nurses, and who takes three-months training on health extension packages. Apart from this, it was also mentioned that different short term trainings were provided for them from different stakeholders.

*“The urban health extension professionals are diploma nurses who were taken three months basis training on the health extension packages. Additionally, they were taken different trainings on specific packages from different stakeholders.” (35–40 years old, male, HEP supervisor`, IDI)*

#### Home visitation

Conducting home-to-home visit was mentioned as a strategy that facilitated the implementation of HEP. It was mentioned that the urban HEPs and HDAs conducted home to home visit to create awareness, promote availability of service, and develop health seeking behavior of community members to utilize the health services, especially related to maternal and child health services. Participants mentioned that one urban HEP visits least 15–20 households every day.

*“The other activity that we conduct is home-to the home visit. Through this activity, together with the health developmental armies, we create awareness and promote the available health service, and support community members to develop the health seeking behavior towards these services. Especially, we conduct the home visit for eligible group of peoples for maternal and child health services.” (35–40 years old, female, HEP, IDI)*

#### Creating model households

In the context of this study, model household refers a family that was trained on the health extension packages from HEPs, and implemented all the packages with the support and close supervision of HEPs. Therefore, the study participants mentioned that awareness creation, and transfer of health knowledge and skill was being done to all household members, and ‘model households’ were created. Through continuous capacity building activity, these groups of families were used as a strategy to improve the accessibility and quality of health extension packages service utilization.

*“Most of our district community members are graduated as a model family and we use them as a strategy for delivering health extension packages to the community members.” (30–35 years old, male, HEPs supervisor, KII)*

#### Strengthening the HDAs

Study participants mentioned that HDAs were used as a strategy for improving the implementation of the urban HEP. It was mentioned that they played a substantial role in facilitating the service utilization, developing health seeking behavior, mobilizing the communities for healthy activities, conducting social mobilization to influence their communities, monitoring the program implementation, becoming a role models for others, identifying community health problems, and proposing best fit local solutions, and sharing best experiences among the network members as well as community members. Therefore, they mentioned that strengthening the HDAs is important to make them strong enough and sustain their functionality to play a significant role in the success of the program.

*“The health developmental armies created awareness about the packages to the community members, played a role in the acceptance and implementation of the health extension program.”(46 years old, male, community member, FGD)*

It was also mentioned that HDA supported HEPs in the identification and referral of pregnant women, conducting postnatal care follow-up, mobilizing the community for immunization campaigns and health education.

*“We teach women in our community. We, the leaders of the Health Developmental Army, advice and convince pregnant mothers to have a follow-up; organize sanitation campaigns, mobilize the community for immunization campaigns, and arranging the coffee ceremony, we discuss information any health with them.” (35–40 year’s old, female, HDA-L, IDI)*

#### Supervision and reporting

Study participants mentioned that the duty station of urban HEP was either at the health center or District Health Office, and there were around 16 HEPs assigned for one health center catchment population. One supervisor was assigned to supervise 8–12 HEPs, and totally there were around 200 HEPs supervisors in the city. The HEPs compiled daily work and reported weekly to their respective supervisors. The supervisors, in turn, compiled and reported the work HEPs to disease prevention and control core processor focal person on a monthly basis.

*“Our duty station is at either health center or district health office. There are around 16 health extension professionals assigned to each health center catchment. One supervisor was assigned to monitor the activity of 8-12 health extension workers. We compile and report what we have done to our respective supervisor on a weekly basis, and then, they compile and submit our work monthly to district disease prevention and control core process focal.” (30–35 years old, female, HEP, IDI)*

#### Referral & Feedback

Study participants also mentioned that the availability of the referral system in between the HEPs and health center improved the program implementation. It was mentioned that the HEPs and HDAs created awareness, promote the availability of services, change the attitude of the community members and develop health seeking behavior towards utilization of health services. Therefore, the HEPs refer cases to the health center when the condition needs further medical attention.

*“We refer community members who need a further check-up and treatment to the health center. We have a referral form to do that.” (30–35 year’s old, female, HEPs, IDI)*

However, it was mentioned that the feedback given to the HEPs varies from facility to facility, and improper handling of referral forms was observed at some health centers. It was also mentioned that in some cases, the HEPs collect feedback from the health centers or clients, and yet in other sites, the supervisors were charged with managing the collection of the referral feedbacks.

*“Most of the time, we receive feedback from the health center. But, sometimes the referral forms and/or feedback papers might be lost either in the health center or by the referred patient.” (30–35 years old, male, supervisor, KII)*

#### Social and community mobilization

A social and community mobilization was the other strategy used for the successful implementation of the program. It was mentioned that the HEPs and HDAs conducted social and community mobilizations, and empowered the community on the health extension packages for facilitating service utilization.

*“We conduct mobilization activities to build the capacity of our community members on health extension packages. We conducted activities like these for sanitation campaigns and to facilitate the utilization of vaccination, family planning methods, and etc.” (50–55 years, old, female, HAD-L, IDI)*

### Challenges for successful implementation of urban HEP

The study found different challenges that affected the implementation of the urban HEP. Generally, the challenges were categorized under three major themes: Health system related challenges, multisectoral related challenges and community related challenges. Under the health system and community related challenges, there are eleven and four categories, respectively. All of the themes are described below.

### Health system related challenges for the implementation of urban HEP in Addis Ababa

This theme has a description of the health system related challenges that affected the implementation of HEP in the study setting. There is no clear cutup demarcation in between the categories. Hence, there are shared concepts in between the categories. Anyone who can read this paper should have to understand this context.

### HEPs recruitment and training related challenges

Study participants mentioned that the HEPs are clinical nurses in their educational background. They got their diploma from private colleges after taking three-years training. They were recruited from those professionals who did not have a history of employment. Moreover, it was mentioned that they were trained on basis health extension packages for three months before they play a role as a HEP. However, they mentioned that the training was not fully empowered them to fully deliver the service packages to the community as a professional or they did not act as a full public health practitioners.

### Health service package and delivery related challenges

Study participants mentioned that even though the HEPs were expected to deliver all the packages equality, there was inconsistency in delivering the packages to the community. The HEPs had directed the majority of the services to the household. However, they were providing immunizations, family planning, and counseling and testing services through outreach in the community, schools, and youth centers. This inequitable delivery of the services was happening from a lack of clear guidance given to the HEPs at the health posts or community level.

*“We are not providing all the 15 packages of the program to each household, because, most of them do not need all the packages. Therefore, we provide them based on their need and problem” (35–40 years old, female, HEPs, IDI)*

### Competing programs and expectations from higher health offices

HEPs reported that competing programs and expectations from the District Health Office affected conducting activities as per their planned activities. For example, they mentioned

*“We may plan to accomplish certain activities. But, there are competing activities or orders that come from the district health office. For example, when we plan to teach mothers or want to have community conversations, the district health office tells us to do other activities like vaccination campaigns.” (30–35 years old, female, HEPs, IDI)*

### Monitoring and supervision related challenges

The HEPs mentioned that there it was expected to have a weekly meeting with the supervisors, monthly meetings with the health center and quarterly meetings with the district health office to discuss on reports or have future directions. However, there were no meetings conducted regularly. Moreover, the supervisors were fault finders rather than behaving a supportive or problem-solving approaches.

*“Supervision meetings are not always held or conducted in a supportive way. Some of the supervisors are fault finders, an overemphasis on checking of records and registers. There is lack of supportive and problem solving approach.” (25–30 years old, female, HEP, IDI)*

### Logistical support and motivation related challenges

The HEPs mentioned that reaching 500 households or more was difficult, especially for wide catchments. Across the interviews, HEPs mentioned that they expensed an average non-reimbursed 10 Ethiopian Birr daily for travelling to and from the furthest area within their catchment. Otherwise, they walked for more than 40 min between clients to complete their daily task.

*“We travel long distances to complete visiting our catchment area. During daytime, the sun is unbearable or don’t even mention about the rainy season. As you know, the transportation problem in Addis Ababa is increasing from day to day. I pay more than 10 Birr per day from my pocket to visit the clients. They provide us monthly only 343 ETH Birr for transportation and phone bills as a benefit. This is not enough for us or expense more our pocket money.” (30–35 years old, female, HEPs, IDI)*

### Supply chain management related challenges

The HEPs mentioned that no logistics system was there to request, track and refill the supplies and consumables due to the shortage of budget. Due to this problem, there were challenges of predicting the supply and refilling the orders of medical and non-medical supplies and equipment.

*“We have requested several times even the basic ones like an umbrella, gown, tissue paper, soap, but they (Health center or district health office directors) complain budget problem. For example, for Vitamin-A supplementation, we need a towel and a scissor, but we bought ourselves from our pocket money” (30–35 years old, female, HEPs, IDI)*

### Household allocation related challenges

The HEPs and supervisors mentioned that, according to the program, 500 households are assigned for one HEP. However, it was mentioned that one HEP was assigned to more than 600 households at some districts, which made difficult to complete the supervision provided for households on time; challenging the successful implementation of the program.

*“Generally, it is expected that one HEP should be assigned for 500 households, and to visit 15-20 households per day. However, this is not true in our District; given that each HEP is assigned for more than 600 households. It takes around two months for each HEP to conduct home visitation and reach all these households.” (40–45 years old, male, supervisor, KII)*

### HEPs related challenges: shortage of HEPs trained on health extension packages

Study participants mentioned that there were HEPs who had not taken a basic three months pre service training on Health Extension Packages. These professionals conduct activities referring the manual, and consulting those who had taken the training. As it was mentioned by the HEPs, non-trained professionals faced difficulties when they conduct activities related to the packages.

*“There were some health extension professionals that had not taken the three months training. They faced some difficulties while conducting activities.” (40–45 years old, male, supervisor, IDI)*

### HEPs related challenges: dissatisfaction on the benefit they got

The HEPs mentioned that the official training provided for upgrading to a higher educational level was also a source of disappointment for many HEPs. They mentioned that the recruitment criteria and process were not clear, the entrance exam was too difficult or promotion after attending the training or upgrading their educational status was not guaranteed.

*“Our salary and benefits are not attractive enough. In addition, there is a problem of education/training opportunities. For example, diploma holder should have to advance their qualification to degree level. However, there are no such opportunities or the selection process is not clear, or the benefit after attending the training is not attractive.” (30–35 years old, female, HEPs, IDI)*

It was also mentioned that HEPs were burnt out due to an unclear career path, including lack of continuing education, and both financial and non-financial incentive mechanisms that posed a major challenge for the Program. In addition to the dissatisfaction in their salary, it was mentioned that there was a lack of basic materials like umbrella, gown, towel, scissors, office, etc. that are important for their work but, made their job very difficult together. Also, it was mentioned that even if HEPs conduct the expected activities, their rights towards the benefits was not respected.

“Even though the HEPs are clinical nurses, they paid lower than those clinical nurses working at the health center. However, they have more workload and need to be paid more.” (35–40 years old, male, supervisor, KII)

*“The workload is too difficult that no one could understand. When you ask your right, you are not granted, even if you fulfill your duties. For instance, we work in emergencies, be it immunization or epidemics like Acute Watery Diarrhea, but the right of the health extension is not respected like other health professionals.” (30–35 years old, female, HEPs, IDI)*

### HEPs related challenges: workload

Health extension professionals and supervisors mentioned that HEP is the focal point of health programming, and so increasingly, different partners are executing their programs through HEP. These created heavier workloads for HEPs that affected the implementation of HEP. They also mentioned that there was no clear cut principle to whom the HEPs would be responsible for or who supervised or monitored them due to the overloading of tasks from different sectors.

*“Sometimes, we do not know our immediate supervisor. Different sectors give us extra work without considering our main responsibility. For example, the District Health office, the health center, Women and child affairs, District small enterprises and the sub-city health officials assign an additional task, which is extra to our main tasks. Even though, working with different sectors is useful, there is no clear framework on how to work with all of them.” (40–45 years old, male, supervisor, KII)*

### HEPs related challenges: transfer

Deployment of HEPs was also raised as a challenge for the implementation of HEP. It was mentioned that even if the HEPs were recruited from the local community, there were professionals transferred to the other areas due to lack of interest to stay in their community. This affected the implementation of the program.

*“According to the Health Extension Program, the health extension professionals are supposed to be recruited from their home communities. But, this does not apply in most cases. As a result health extension workers do not stay long in the area of their deployment.” (30–35 years old, female, supervisor, IDI)*

### Multisectoral collaboration related challenges for the implementation of HEP

Study participants mentioned that there was poor multi-sectoral collaboration resulting in diminished work results of health extension professionals.

*“There is a need to work in collaboration with different sectors, given that we alone couldn’t solve the community’s problem. For example, some households do not have latrines, solid waste collection or a sewerage line problem. Therefore, unless we work with other governmental and non-governmental sectors, it is difficult for us to solve these problems.” (30–35 years old, female, HEPs, IDI)*

### Community-related challenges that affected the implementation of HEP

#### Economic constraint

The study participants mentioned that the community members had an economic problem to change into practice the support they obtained from HEPs. In addition, they indicated that most of the problem existed within the community need involvement of different private and public stakeholders.

*“The health education professionals educate us to have latrines, constructing sewerage lines and environmental hygiene. But, we have no space or money for latrine construction. We barely fulfill our daily basic needs. We need support from the government and private companies.” (35–40 years old, female, community member, FGD)*

#### Model household related challenges: lack of graduating as per the plan

The HEPs mentioned that there were additional challenges they had experienced from the community. It was mentioned that model households were expected to graduate within four months of training. However, they did not graduate as per the plan, given that the training schedule was dependent on the family’s willingness.

*“The model households are expected to graduate within four months. However, since the provision of training depends on household’s schedule, they do not graduate as per our plan.” (30–35 year’s old, female, HEPs, IDI)*

#### Health developmental army related challenges: need of incentive from health facilities

Study participants mentioned that the HDAs voluntarily played a significant role to change their community to seek health care. However, there was no recognition or compensation given them from the district health office or health center. Therefore, they mentioned that for the sustainability of the program, there should be some material compensation provided to them for extra services they rendered to communities.

*“There is no support we receive from the District or the health center. I am doing this voluntarily, because, I would like to see the change of my community. It will be great if we receive recognition either from the District or the Health center.” (35–40 years old, female, HDAL, IDI)*

#### Urbanization and change of residency

The HEPs mentioned that mobility of community members has been challenged them to identify and to provide health services to in a consistent and continual manner. For example, they mentioned that while they conduct home visitation to provide continuous training and service, there were community members who changed their residence, which would be a major challenge even for the future. This is because they mentioned that the target populations’ move from place to place across towns or in the same town which affected the continuity of services being provided to the clients.

*“The nature of the program needs to conduct home to home visitation and provision of continuous training on the packages. However, there were community members who move from one area to the other within the city, and become difficult for us conduct home visitation and training.” (30–35 years old, female, HEPs, IDI)*

## Discussion

This study found that the 15 health service packages were provided based on household need. The strategies used for the program implementation were the provision of training to HEPs and HDAs, home visitation, creation of model households, strengthening of HDAs, supervision and reporting, referral and feedback, and social and community mobilization. However, the implementation of the program was affected by health system, multisectoral and community related challenges.

From this study, it was found that all 15 essential health service packages were delivered to the community members. However, all the packages were not necessarily provided to each community members, rather it depends on the household need. Importantly, this is good to save time and for the successful implementation of the program. Similarly, the finding of this study was supported by the findings of other studies conducted in different context [5, 10].

The study found that implementation of the HEP was facilitated through providing basic and continuous training and education for HEPs. In contrast, this study also identified that there were HEPs who had no trained on the health extension packages or had not given a chance to upgrade their educational status, and affected the implementation of the program. However, the HEPs who served for a minimum of two years and passed the competency assessment will be trained for one year to upgrade to the next level [11]. Therefore, this calls for action to put this rule and regulation into practice to make this professional equipped with knowledge and skill, additionally, to motivate them through the developmental carrier structure for better program implementation. Similarly, inconsistent pre-service training and lack of regular and standard training materials for on the job training /refresher training were mentioned on another study that affected the program implementation [5].

Creating a model household was also found used as a strategy to foster implementation of the program. It was indicated that there were graduated model families that transfer knowledge or skill, and become a role model for the community to improve the program implementation. This implies that households are essential for facilitating the delivery of the health extension packages to improve the program implementation. Similarly, a study conducted in different settings indicated that successful health service package utilization was achieved through the creation of model households [5, 10, 12, 13]. However, this study also explored that model households were not graduated as per the HEPs plan due to community related challenges. Therefore, there is a need to convince community members to give their emphasis on it, and contribute to the improvement of community members’ health through facilitation of the program implementation.

Home visitation was also one of the strategies used for successful program implementation. However, HEPs were not conducted home to home visit to the households on a regular basis due to the workload from competing interests and expectations from different stakeholders, including from the district health office. Also, it was affected that more households were assigned to one HEP, which was above the expectation [5]. Therefore, stakeholders, including the district health office and health centers, should have to support the HEPs and work together with them rather than ordering to do additional tasks and requesting reports. On the other hand, there is a need to deploy the number of HEPs as per the catchment population.

The urban HEP needs strong involvement of the HDAs to promote health and create demand for health services, identify locally salient bottlenecks that hinder uptake of services, and scale up best practices [5]. This study also found that the HDAs played a significant role in program implementation, and strengthening them was used as a strategy. Therefore, this implies that HDAs are the most important agents for the creating awareness, promoting the health extension packages, and developing health seeking behavior of the community members. However, this study also explored that the activities conducted by the HDAs were affected, given that they conducted activities voluntarily or there were no incentives (i.e., motivation, material compensation, and etc.) given to them from health sectors and others. Therefore, this underscores a need to motivate the HDAs through providing appreciation and material compensation, if possible, and they should have to be convinced about their importance for the role they would play in improving the health of the community.

Monitoring or supervision provided to the HEPs was also facilitated the implementation of the program in the city. However, it was also mentioned that the supervision or monitoring was not conducted on a regular basis or the supervisors had a faultfinding behavior rather than building the capability of HEPs. Similarly, this finding was supported by another study which indicated that inadequate supportive supervision and management was a barrier or the implementation of HEP [14]. Therefore, this underscores the need to provide a regular supportive supervision or monitoring to the HEPs to build their capacity, and improve the implementation of the program.

Social and community mobilization was also identified as a strategy for the successful implementation of the HEP used by the HEPs in the city. Similarly, it was mentioned that for the successful implementation of the HEP, active community participation is needed [7], and it is believed that community mobilization improves communities’ uptake of the program [14]. Therefore, the HEPs with the HDAs should have to conduct a continuous a social and community mobilization to empower the community and sustain the program for improving the health of the community.

The other challenge that was explored in this study and affected the implementation of HEP is a supply chain management. The study found that there HEPs faced problems of requesting, tracking and re-filling medical supplies due to lack of logistics systems. This implies that there was a poor supply chain management in between the HEPs and health sectors (district health office and health office). This underscores the importance of strengthening the existing supply chain management and fulfills all the required materials used for managing the stocks out and stock in medical supplies and equipment’s. Similarly, the poor supply management system was mentioned as a factor in the program implementation on previous study [14].

This study also found that HEPs were not satisfied with the support or benefit they were obtained, load of the tasks assigned from different stakeholders, and education access for upgrading their status. This dissatisfaction challenged the successful implementation of the program. Therefore, there should be a need to stick to the policy issues developed to keep the benefit of these professionals, and stakeholders should have support and work with them rather than ordering and requesting reports. Similarly, this finding was supported with other study [14].

The health systems are social institutions, with chains of relationships between different actors, and optimal performance depends on the strength and nature of relationships between all actors [15]. In contrast, this study found that there was a lack of multisectoral collaboration which challenged the successful implementation of the program. However, different sectors did not support the HEPs for program implementation; rather load different tasks to these professionals. Therefore, each and every sector should have to support and work with HEPs successful implementation of the program. This finding was supported by the finding of other studies which indicated that lack of coordination between the health extension professionals and other sectors such as agriculture, water and sanitation and education programs posed problems to the health extension program [5, 14].

### Strengths and limitations

This study has several strengths. To the best knowledge of the authors, this is the first study conducted in Ethiopia that explored the implementation, experience, and challenges of Urban Health Extension Program. A diversified group of participants was involved in the study in order to generate ideas from different perspectives. The focused groups allowed frank discussion among attendees. The findings of this study significantly help local planners and managers to improve implementation of UHEP. This study also has limitations. There might be the probability of social desirability bias.

## Conclusions

From this study, it was understood that the health extension packages were delivered to the community members based on the need of households, and the program has made a significant contribution to the health of community through creating demand and increasing access to health services, and strengthening the referral system. The study also explored different strategies used to implement the program. However, the strategies were not strongly put into practice and challenged the program implementation. Therefore, there is a need for strengthening and sustaining the strategies, developing different strategies and taking actions to address such challenges for successful implementation of the HEP in the city.

The health center and district health office should have to deploy the required number of HEPs, regularly supervise and monitor the HEPs, and put into practice the existing rules and regulations developed to ensure the benefit of the HEPs in terms of continuous education and training and promotion. Health care providers, HEPs, and HDAs should have to conduct SBCC interventions to change the perception of the community members on the program; strengthen and sustain the model households that play a role of awareness creation, service promotion, and develop the health seeking behavior of the community members. Stakeholders from different sectors should support and work together with the HEPs. Health sectors should have to strength the existing supply chain management and fulfill all the required materials used to manage the medical supplies and equipment’s.

The national and local program planners and managers are advised to give due emphasis to the implementation of UHEP; solve the bottlenecks and reward those who successfully achieved their performance. In addition, inter-sectoral collaboration should also be emphasized. The current study was conducted only in Addis Ababa. Therefore, researchers are recommended to conduct studies on this issue broadly as regional and national.

## Supplementary Information

**Additional file 1: Supplementary file 1.** Guide for data collection (English version). The data contain an English version of interview and Focus group discussion guides developed and used for this study.

## Data Availability

All data generated or analyzed during this study are included in this published article.

## Abbreviations

FGD: Focused Group Discussion
HC: Health Center
HDA: Health Developmental Army
HEP: Health Extension Professional
HRD: Human Resource Development
IDI: In-depth Interview
KII: Key Informant Interview
NGO: Non-governmental Organization
PhD: Philosophical degree
UHEP: Urban Health Extension Program
WHO: World Health Organization
