# Supplementary material for: Implementation, experience, and challenges of urban health extension program in Addis Ababa: a case study from Ethiopia
Source: BMC Public Health. 2021 Jan 19;21:167. doi: 10.1186/s12889-021-10221-0 (PMC7816480; doi:10.1186/s12889-021-10221-0)
Supplement: Supplementary file 1 — Additional file 1: Supplementary file 1. Guide for data collection (English version). The data contain an English version of interview and Focus group discussion guides developed and used for this study. [file 12889_2021_10221_MOESM1_ESM.docx]

**Guide for data collection (English version)**

Name of Sub-city: ___________________ District: ______________________

Name of data collector: _____________________________________________

Date of interview: _____ /____/ _____

Time start:__/__:__/___ time end:__/__:____

Place of interveiwe______________________________

**Part I: Interview guide for the Urban health extension professionals, supervisors, health developmental armies and Addis Ababa City HEP administrator**

1. Socio-demographic related questions:-
2. How old are you?
3. Please would you tell me your educational status?
4. Please would you tell me your marital status?
5. Please would you tell me number of children you have?
6. Please would you tell me your occupation?
7. Please would you tell me your profession?
8. Please would you tell me your ethnicity?
9. Please would you tell me your religion?
10. Please would you tell about the urban health extension program?
11. How do you see the implementation of the urban health extension program?
12. Please would you tell me the strategies used for the implementation of the urban health extension program?
13. Please would you tell me the experiances you have on the implementation of the urban health extension program?
14. Please would you tell me the challenges for the successful implementation of the urban health extension program?
15. Please would you tell me the suggestions you have on the program implementation?
16. Please would you summarize key points from our discussion?

**Part II: FOCUS GROUP GUIDE: For community members**

1. Please would you tell me anything you know about the urban health Extension Program?
2. Please would you tell me about the urban health extension packages?
3. How do you see the implementation of the program?
4. How does the program implemented in your catchement?
5. How do you see the acceptability and service uptake of the program?
6. Please would you tell me the benefits you gave gained from the program?
7. Please would you tell me the challeneges for the implementation of the program?
8. Please would you tell me the suggestions you have on the program implementation?
9. Please would you summarize key points from our discussion?
